# Evaluation of the antibacterial activities of face masks coated with titanium dioxide nanoparticles

**DOI:** 10.1038/s41598-022-23615-w

**Published:** 2022-11-04

**Authors:** Omar B. Ahmed, Turki Alamro

**Affiliations:** 1 Department of Environmental and Health Research, The Custodian of the Two Holy Mosques Institute for Hajj and Umrah Research, Umm Al-Qura University, Makkah, Saudi Arabia; 2 Department of Mechanical Engineering, College of Engineering and Islamic Architecture, Umm Al-Qura University, Makkah, 24372 Saudi Arabia

**Keywords:** Biotechnology, Microbiology, Health care, Materials science

## Abstract

To control infectious diseases, various applications of nanotechnology have been used to enhance the self-cleaning and antibacterial properties of materials**.** This study aimed to evaluate the antibacterial properties of face masks coated with TiO2 nanoparticles**.** The antibacterial efficacies of cloth face masks coated with TiO2 were measured by inoculating them in bacterial suspensions (10^5^ CFUs from both *E. coli* and *S. aureus*). The results showed that TiO2 nanoparticle solutions (at 2%) reduced the starting inoculum of 10^5^ CFUs (5 log cfu/cm^2^) of *E. coli* and *S. aureus* to 1.3 and 1.68 log, respectively, with antibacterial activities of 3.7 and 3.34 log, respectively. Furthermore, at a 1% concentration, the antibacterial activities against *E. coli* and *S. aureus* were 2.1 and 2.01 log, respectively, while at a low concentration (0.5%), the antibacterial activities against *E. coli* and *S. aureus* were 1.8 and 1.72 log, respectively. The CFUs in all the experimental groups were significantly lower than those in the control group (saline). In conclusion, TiO2 nanoparticle solutions with a high concentration (2%) demonstrated a strong antibacterial effect on *E. coli* and *S. aureus*, and the difference was statistically significant, while a significant antibacterial activity was demonstrated with lower concentration (0.5% and 1%) nanoparticle solutions of TiO2 after 18 h. There was a statistically significant difference regarding colony reduction between *E. coli* and *S. aureus* even at 3 h. The antibacterial activities of TiO2 in face masks could be promising for reducing the risk of bacterial infections.

## Introduction

The development of nanotechnology is a promising technological trend that may have a large impact in many fields, such as physics and biology, medicine, electronics, food, water quality, the textile industry, air quality and biomechanics ^1^. It is defined as “a science and technology which is conducted at one billionth (10^−9^) part of metre,” i.e., on the nanoscale (1–100 nm).

There are many types of nanoparticles, such as metallic, nonmetallic, organic, and inorganic nanoparticles ^2^. Titanium, copper, and silver nanoparticles are examples of metallic nanoparticles. Titanium dioxide (TiO2) has unique properties, such as a low cost, stability, low toxicity, high refractive index, high optical properties, high ultraviolet absorbance, strong redox ability, high energy gap (i.e., 3.2–5.2 eV), and has good electrical, optical and magnetic properties ^3,4^. It is necessary to fully define the characteristics of nanoparticles, such as their size, shape, surface morphology, crystallinity, and light absorption, using appropriate characterization techniques ^5^, such as microscopy techniques (electron microscopy or scanning probe microscopy). Additionally, optical techniques (spectroscopy) can be used to study nanoparticle characteristics, such as reflectance, transmittance, photochemistry, and luminescence ^6^. Brunauer–Emmett–Teller (BET), X-ray diffractometry (XRD), and infrared spectroscopy (IR) are the most extensively used techniques for the characterization of NP structures and may be used to describe the phase, particle size, type, and crystal nature of nanoparticles. The surface quality of nanoparticles is highly influenced by their mechanical properties, which include stress, surface coatings, hardness, strain, friction, and adhesiveness. The characteristics of TiO2 include stability, low cost, nontoxicity, biocompatibility, optical, and electrical properties. It mostly appears in three distinct forms, including brookite, anatase, and rutile, with different structures. Thermodynamic simulations show that during heating, both anatase and brookite transform into rutile, which is more stable at all temperatures and pressures below 60 kbar ^7^. Nanomaterials, such as TiO2 photocatalysts, have demonstrated remarkable activity in the photodegradation of a variety of organic and inorganic pollutants. Since organic contaminants may totally degrade into harmless materials under normal circumstances of temperature and pressure, it is expected that photocatalysis will soon be one of the most efficient methods for dealing with diverse types of contaminants. Pollutants, including herbicides, carboxylic acids, and alcohols, can be entirely broken down into carbon dioxide, water, and simple minerals ^8^. The photocatalyst has to have specific qualities, such as the right particle size, shape, crystallinity, and anatase to rutile ratio, to be particularly effective. The most commonly used methods for producing TiO2 nanoparticles are electrodeposition, reverse micelles, the sol–gel method, metal organic chemical vapour deposition, the flame combustion method, gas phase (aerosol) synthesis, hydrothermal methods, wet-chemical synthesis by the precipitation of hydroxides from salts, and microemulsion-mediated methods ^9^. The sol–gel process is a wet-chemical technique that is mostly used in the fields of materials science and ceramic engineering. It can be defined as the conversion of a precursor solution into an inorganic solid through polymerization reactions induced by water ^10^. Hydrolysis forms a sol that is basically a dispersion of colloidal particles in a liquid, and condensation leads to the formation of a gel. Compared to the methods discussed above, the sol–gel process is very promising for the synthesis and preparation of inorganic and organic‒inorganic hybrid nanomaterials because it allows the use of low processing temperatures (< 100 °C) and molecular level composition homogeneity ^10^. Particle size and shape are easy to control using the sol–gel method. The sol–gel process produces fine, spherical powders of uniform size and has been widely used to synthesize TiO_2_ materials and normally proceeds via an acid-catalysed step of titanium (IV) alkoxides ^11^. One of the most attractive features of the sol–gel process is the possibility of shaping the resulting material into desired forms, such as fibre, film and monodispersed powder. Several steps and conditions are applied in a sol–gel process to control the final morphology, as suggested by Mehrotra and Singh ^10^. The use of TiO2 as a photocatalyst to kill microorganisms has been known for a long time ^12^. The antibacterial properties and mechanisms of nanotechnology have been widely discussed, including those of nanoparticles of TiO2, which have been extensively applied due to their photocatalytic properties to breakdown and remove dirt, odour, and kill bacteria. The mechanism of this technique depends on the generation of reactive superoxide radicals (O2^−^ and ·OH) on the surface of TiO2 molecules during the process of photocatalysis when exposed to light of an appropriate wavelength ^13–15^. Oxygen radicles affect bacterial cells by different mechanisms, leading to their death. Both types of bacteria differ from each other in their response to antibacterial nanoparticles. Disinfection is defined as the treatment procedure used to eliminate pathogenic microorganisms, but it may not eliminate bacterial spores ^16^. During recent decades, TiO2 in the form of nanoparticles has been known to have broad-spectrum antibacterial activities ^17,18^. Fabric face masks are materials that are used to protect against breathable pathogens (bacterial or viral) ^19^. They are classified as *full* masks, *half masks, and quarter masks.* The filtering efficiency *of* face masks varies from one to another depending on the density of the face mask material ^20^. With the continuous use of face masks without regular exchange, improper washing can potentially contaminate surfaces, as temperature and humidity induce moisture and hence microbial colonization; in addition, improper use may lead to the risk of pathogen spreading ^21–25^. The disposal of face masks has led to an enormous increase in waste, which is classified as “hazardous with infectious risk”, and face masks are disposed of as biological hazards ^26^. Nanoparticles have been shown to be capable of killing a wide range of organisms, including gram-negative and gram-positive bacteria, which differ in regard to their cellular wall and envelope and hence their resistance to disinfectants ^27^. In addition, many other organisms, including viruses, fungi, algae and protozoa, have been shown to be killed by TiO2 nanoparticles ^12^. It has been shown that these nanoparticles are useful for the disinfection of face masks ^16,17^. Face masks coated with TiO2 are widely applied for enhanced self-cleaning and antibacterial properties to control infectious diseases, such as COVID-19 ^28^. This paper aimed to evaluate the antibacterial properties of face masks coated with TiO2 nanoparticles.

## Materials and methods

### Sol–gel preparation of TiO2 nanoparticles

TiO2 nanoparticle solution was prepared by the hydrolysis and condensation of 97% titanium tetraisopropoxide in an acidic aqueous solution (low pH) of glacial acetic acid and 37% HCL acid with different concentrations of TiO2 precursor. The mixture was heated at 60 °C under vigorous stirring for 90 min. TiO2 nanoparticle powder was used to impart self-cleaning and antibacterial properties.

### Bactericidal efficacy against contaminated mask surfaces

Five pieces of fabric face mask were selected for this study, each composed of 80% polyamide and 20% elastane. The antibacterial activities of these face masks were evaluated with suspension tests according to the standard ISO 20743:2021 entitled “Textiles—determination of antibacterial activity of antibacterial finished products.”

To test the biocidal activity, two types of bacteria were used, one gram-positive bacteria and one gram-negative bacteria.

All masks were coated with a suspension of TiO_2_ nanoparticles at different concentrations of 0.5, 1 and 2% *w*/*w*, dried for approximately 24 h and cut into pieces of approximately 2 × 2 cm. Two bacterial suspensions of *E. coli* (ATCC 25922) and *S. aureus* (ATCC 25923) were cultured on tryptic soy agar (Oxoid, UK) and incubated at 35 °C overnight. Each coated mask piece was inoculated with a suspension of McFarland standard (1.0 × 10^5^) colony forming units (CFUs/ml) of *E. coli* and *S. aureus* at time 0 (T0). The swabbing (2 × 2 cm) of each mask piece was performed at 7 equal intervals for analysis at 0, 3, 6, 9, 12, and 15 and at 18 h. To determine the colony count of each piece, the swabs were diluted in one millilitre of sterile nutrient broth in tubes. The whole suspension of each of these tubes was drawn and spread over nutrient agar to determine the colony count with a digital colony counter. The mean value of CFUs and the antibacterial activities were obtained every three hours (at 0, 3, 6, 9, 12, 15, and 18 h), and the number of CFUs was reported as CFU/cm^2^. The colony plate count method was used for the enumeration of bacterial CFUs after overnight incubation at 37 °C. All tests were run in triplicate. Another five pieces were kept as controls by using saline solution (0.85% NaCl) instead of TiO2 nanoparticles. The antibacterial activities were calculated according to the formula below ^29^, while the assessment was performed according to ISO 20743-2021 (Table 1).

$${\text{Antibacterial}}\,{\text{activity }}\left( {{\text{reduction}}} \right)\, = \,{\text{Log1}}0{\text{CFU}}\,\left( {{\text{negative}}\,{\text{control}}} \right) - {\text{Log1}}0{\text{CFU}}\,({\text{sample}}).$$

**Table 1:** Assessment guide of antibacterial activities (reduction) of face masks coated with TiO2 nanoparticles.

Reduction efficacy	Indication	References
Less than 0.5	No antibacterial activity	^30,31^
Equal or more than 0.5 and less than 1	Slight antibacterial activity	
More than 1 and less than 3	Significant antibacterial activity	
Equal or more than 3	Strong antibacterial activity	

One-way analysis of variance (ANOVA) was used to check the mean differences among the antibacterial activities at different time intervals within groups and at different concentrations of TiO2 (*P* < 0.05). The paired *t* test was used to check the mean differences among the antibacterial activities (reduction) at different time intervals within groups (*P* < 0.05). Statistical analyses were performed using SPSS-25 (Inc., IBM, Chicago, IL, USA).

## Results

The present study evaluated the antibacterial activities of mask samples using TiO2 nanoparticles as antibacterial agents against *E. coli* and *S. aureus*. Table 2 shows the bacterial count log (log cfu/cm^2^) at different TiO2 concentrations. When TiO2 nanoparticle solutions of a high concentration (2%) were used, the starting inoculum of 5 log (10^5^ cfu/ml) bacteria was reduced to 1.3 and 1.68 log for *E. coli* and *S. aureus,* respectively. When TiO2 nanoparticle solutions of a low concentration (0.5%) were used, the starting inoculum of 5 log bacteria was reduced to 3.2 and 3.3 log for *E. coli* and *S. aureus,* respectively. When TiO2 nanoparticle solutions were used at a concentration of 1%, the starting inoculum of 5 log bacteria was reduced to 2.9 and 3.01 log *E. coli* and *S. aureus*, respectively. Table 3 shows the bacterial count (log cfu/cm^2^) of the negative control after exposure to normal saline instead of TiO_2_ solution. It is noted that the starting inoculum of 5 log of both *E. coli* and *S. aureus* remained unchanged or slightly increased.

**Table 2:** Mean bacterial count after exposure to different TiO2 concentrations.

Time (h)	TiO2% (*w*/*w)*					
	0.5	1.0	2.0	0.5	1.0	2.0
T_0_	5	5	5	5	5	5
T_3_	4.2	3.96	3.74	4.3	3.99	3.81
T_6_	4.1	3.79	3.56	4.2	3.9	3.78
T_9_	4.0	3.7	3.03	4.1	3.79	3.1
T_12_	3.85	3.49	2.5	3.9	3.63	2.6
T_15_	3.6	3.04	2.1	3.7	3.35	2.15
T_18_	3.2	2.9	1.3	3.3	3.01	1.68
	*E. coli* count (log)			*S. aureus* count (log)

**Table 3:** Bacterial count (log cfu/cm^2^) after exposure to saline solution (negative control).

Time (h)	NaCl (0.85%)	
T_0_	5	5
T_3_	5.02	5.07
T_6_	5.02	5.05
T_9_	5.01	5.03
T_12_	5	5.03
T_15_	5.04	5
T_18_	5	5.02
	*E. coli* count (log)	*S. aureus* count (log)

**Figure 1:**
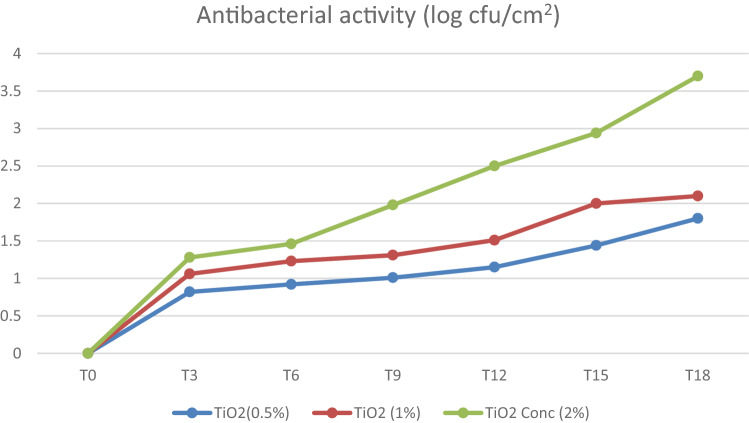
Antibacterial activity of TiO2 nanoparticles against *E. coli.*

**Figure 2:**
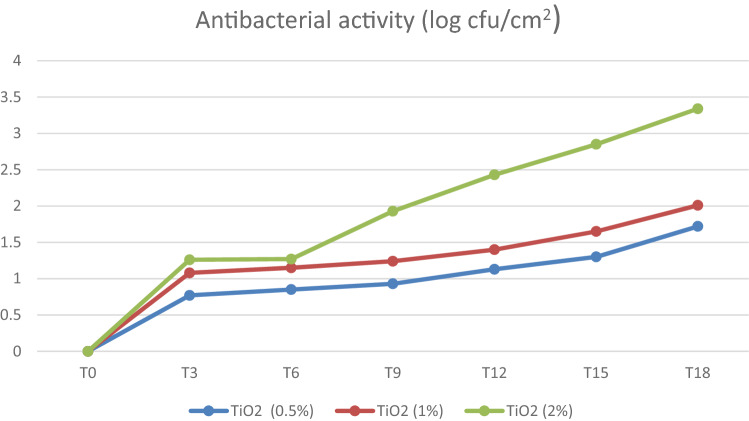
Antibacterial activity of TiO2 nanoparticles against *S. aureus.*

Table 4, Figs. 1 and 2 show the significant antibacterial efficacy of the TiO2 nanoparticles applied to face masks against *E. coli* and *S. aureus* depending on the concentration. When TiO2 nanoparticle solutions of a high concentration (2%) were applied to face masks against *E. coli* and *S. aureus*, efficacies of 3.7 and 3.34 log reduction were obtained, respectively, which is considered strong antibacterial activity (Table 1). Furthermore, at a 1% concentration, the efficacy against *E. coli* and *S. aureus* was 2.1 and 2.01 log reduction, respectively, which is considered a significant antibacterial activity (Table 1), while at a low concentration (0.5%), the efficacy against *E. coli* and *S. aureus* was 1.8 and 1.72 log reduction, respectively, which is also considered a significant antibacterial activity (Table 1).

**Table 4:** Antibacterial activity (reduction) after exposure to different TiO2 concentrations.

Time (h)	TiO2% (*w*/*w)*					
	0.5	1.0	2.0	0.5	1.0	2.0
T_0_	0	0	0	0	0	0
T_3_	0.82	1.06	1.28	0.77	1.08	1.26
T_6_	0.92	1.23	1.46	0.85	1.15	1.27
T_9_	1.01	1.31	1.98	0.93	1.24	1.93
T_12_	1.15	1.51	2.5	1.13	1.4	2.43
T_15_	1.44	2	2.94	1.3	1.65	2.85
T_18_	1.8	2.1	3.7	1.72	2.01	3.34
	*E. coli* (log reduction)			*S. aureus* (log reduction)

The CFUs of all the experimental groups were significantly lower in comparison with those of the control group (Saline). TiO2 nanoparticle solutions of a high concentration demonstrated better antimicrobial efficacy. There was a significant difference among the antibacterial activities at different time intervals (*P* = 0.00), while there was no significant difference among the antibacterial activities at TiO2 concentrations within the groups (*P* = 0.184). There was a significant difference in the data between *E. coli* and *S. aureus* (*P* < 0.05).

## Discussion

This study reports the antibacterial effect of TiO_2_ nanoparticle materials against bacterial strains in face masks contaminated with *E. coli* and *S. aureus* after exposure to different concentrations. TiO_2_ nanoparticles were chosen because they act as a photocatalyst to kill microorganisms. In addition, TiO2 has a low cost, stability, low toxicity, high ultraviolet absorbance, and a high energy gap (i.e., 3.2–5.2 eV) ^3,4^.

This study reports the antibacterial effect of TiO_2_ nanoparticle materials against bacterial strains in face masks contaminated with *E. coli* and *S. aureus* after exposure to different concentrations. Results were collected for TiO2 nanoparticle solutions of different concentrations (0.5–2%). A higher concentration reduced the starting inoculum from 5 to 1.3 and 1.68 log for *E. coli* and *S. aureus,* respectively, while a lower concentration (0.5%) reduced the starting inoculum from 5 to 3.2 and 3.3 log for *E. coli* and *S. aureus,* respectively. When TiO2 nanoparticle solutions were used at a concentration of 1%, the starting inoculum was reduced from 5 to 2.9 and 3.01 log *E. coli* and *S. aureus,* respectively. The CFUs in all the experimental groups were significantly lower in comparison with the control group (Saline). TiO_2_ nanoparticles have been tested for their antibacterial efficacy in different matrices, such as in fabrics of various materials ^32^.

Gram-positive bacteria (*S. aureus*) have one membrane surrounded by a very thick wall made of peptidoglycan. Gram-negative bacteria (Escherichia coli (*E. coli*)) have a very thin membrane, which constitutes a barrier that retains the toxic agent, while the wall has been reported to be sensitive to the peroxidation caused by TiO_2_^33^.

A great majority of studies have been performed with gram-negative and gram-positive bacteria ^12^. TiO2 nanoparticle-treated materials have been shown to be effective against bacteria and have been reported to have excellent disinfectant properties against other types of microbial contamination ^34^. The disinfectant property of TiO2 nanoparticles is greatly dependent on the photocatalytic behaviour of TiO2 ^35,36^.

Sunada et al. ^37^ suggested that the cell wall of *E. coli* cells acts as a barrier to the mechanism process because the outer membrane serves as a barrier, and the outer membrane decomposes first before the complete decomposition of entire cells. The photocatalytic oxidation mechanism of TiO2 nanoparticles was studied by Nadtochenko et al. ^38^, who demonstrated that the organic material content is oxidized due to photocatalytic activity, which leads to TiO_2_ surface cleaning, and consequently, the organic material of the cell wall membrane reduces holes in the TiO_2_ valence band. Our results showed a significant antibacterial efficacy (log reduction) of the TiO2 nanoparticles in the face masks depending on the concentration. When TiO2 nanoparticle solutions of a high concentration (2%) were applied to face masks against *E. coli* and *S. aureus*, efficacies of 3.7 and 3.34 log reduction were obtained, respectively, which is considered strong antibacterial activity (Table 1). Furthermore, at a 1% concentration, the efficacy against *E. coli* and *S. aureus* was 2.1 and 2.01 log reduction, respectively, which is considered a significant antibacterial activity (Table 1), while at a low concentration (0.5%), the efficacy against *E. coli* and *S. aureus* was 1.8 and 1.72 log reduction, respectively, which is also considered a significant antibacterial activity (Table 1). TiO2 nanoparticle solutions of a high concentration demonstrated better antimicrobial efficacy (*P* < 0.05). There was a significant difference among the antibacterial activities at different time intervals within the groups. There was also a significant difference in the data collected for *E. coli* and *S. aureus* (*P* < 0.05).

Our results are similar to earlier findings by some authors ^39,40^ who showed a reduction in bacterial count after contact with TiO2 nanoparticles.

Another finding showed significant antibacterial activities after two and four hours, suggesting that the formulation improved and increased efficiency ^41^. Initially, the colony count fell rapidly with increasing concentrations of TiO_2_, but with 2% TiO2, the colony count was reduced more effectively. It was reported that the bactericidal effect induced by TiO2 nanoparticles depends on the time, concentration and light intensity ^40,42–44^. The different antibacterial activities of TiO2 nanoparticles on *E. coli* and *S. aureus* are probably due to the differences in the bacterial cell wall structures. *S. aureus* has only a plasma membrane and possesses a thick peptidoglycan layer, while *E. coli* has a thin cell wall composed of two cell membranes ^45^. Our result is similar to other findings conducted by different researchers who reported the antibacterial activities of other nanomaterials ^46,47^. In a similar study on facemasks, a 100% reduction in *E. coli* and *S. aureus* with minimum inhibitory concentrations of 1/128 and 1/512, respectively, was reported ^48^. Gogniat et al. ^49^ found that the rate of cell killing was positively correlated with the bactericidal effect of TiO_2_ and the aggregation of TiO_2_, which led to membrane integrity. Various studies report different TiO_2_ concentrations and antibacterial activities ^50^. The inactivation process of bacteria increased as the exposure time increased, and sterilization efficiency increased. This also is in agreement with other reports that deal with the effect of TiO2 on bacterial cells ^37,51^. Caballero et al., studied the inactivation of *E. coli* and found that the inactivation rate of *E. coli* increased with decreasing TiO_2_ concentration. They also showed that increasing particle contact with the bacteria enhanced the disinfection process and that excess TiO_2_ might not enhance the antibacterial effect ^51^. In a similar study, Margarucci et al. ^21^ reported a significant reduction in the microbial load (over 90%) in facemasks using both *E. coli* and *S. aureus* bacteria within less than 1 h.

The present study was undertaken in Makkah city, Saudi Arabia, which is considered the location of one of the world’s largest annual mass gatherings; hence, respiratory diseases are a major concern.

Public health organizations advise those on pilgrimages to use face masks since they are known to stop the transmission of respiratory illnesses from one person to another. Bacterial growth may result from the repeated usage of facemasks, breathing, and saliva aerosols. In addition, the disposal of face masks might result in a significant rise in waste that is considered a “hazardous contagious risk” because of the crowds and mass gatherings. Reusable face masks with antimicrobial effects would therefore be quite helpful. This study’s findings will further reveal how respiratory diseases can be controlled during mass gatherings. These findings are of major importance to assessing how TiO2 nanoparticles can be a major antimicrobial agent, especially when exposed to visible light, whereby they absorb light and act as photocatalysts that successfully kill *S. aureus* and *E. coli*. The antibacterial activities of face masks coated with titanium dioxide nanoparticles would lead to environmental sustainability in different occupational or recreational settings.

In conclusion, the CFUs in all the experimental groups were significantly lower than those in the control group (saline). TiO2 nanoparticle solutions of a high concentration demonstrated better antimicrobial efficacy, and the difference was statistically significant. Strong antibacterial activity was demonstrated against *E. coli* and *S. aureus* by face masks coated with nanoparticle solutions of a high concentration (2%), while significant antibacterial activity was demonstrated using both 0.5% and 1% TiO2 nanoparticle solutions after 18 h. There was a statistically significant difference regarding colony reduction between *E. coli* and *S. aureus* even at 3 h. The antibacterial activities of TiO2 in face masks could be promising in reducing the risk of bacterial infections.

## Supplementary Information

Supplementary Information.

## Data Availability

All data generated or analysed during this study are included in this published article [and its supplementary information files].
